# Infection and death by COVID-19 in a cohort of healthcare workers in Mexico

**DOI:** 10.5271/sjweh.3970

**Published:** 2021-06-29

**Authors:** Eduardo Robles-Pérez, Belinda González-Díaz, Maximino Miranda-García, Victor Hugo Borja-Aburto

**Affiliations:** Dirección de Prestaciones Médicas, Instituto Mexicano del Seguro Social, Ciudad de México, México; Coordinación de Vigilancia Epidemiológica, Instituto Mexicano del Seguro Social, Ciudad de México, México

**Keywords:** death due to COVID-19, health worker, occupational risk, SARS-Cov2

## Abstract

**Objective:**

This study aimed to estimate the risk of SARS-Cov2 infection and severe COVID-19 among healthcare workers from a major social security system.

**Methods:**

This study actively followed a cohort of social security workers from March to December 2020 to determine the number of laboratory-confirmed symptomatic cases, asymptomatic associated contacts and COVID-19-associated hospitalizations and deaths. Workers were classified into those providing direct care to infected patients (COVID teams), other active healthcare workers (OAHCW), and workers under home protection (HPW). The number of cases and rates were also estimated by job category.

**Results:**

Among a total of 542 381 workers, 41 461 were granted stay-at-home protection due to advanced age or comorbidities. Among the 500 920 total active workers, 85 477 and 283 884 were classified into COVID teams and OAHCW, respectively. Infection rates for COVID teams, OAHCW, and HPW were 20.1% [95% confidence interval (CI) 19.8–20.4], 13.7% (95% CI 13.5–13.8), and 12.2% (95% CI 11.8–12.5), respectively. The risk of hospitalization was higher among HPW. COVID teams had lower mortality rate per 10 000 workers compared to HPW (5.0, 95% CI 4.0–7.0 versus 18.1, 95% CI 14.0–23.0). Compared to administrative workers, ambulance personnel (RR 1.20; 95% CI 1.09–1.32), social workers (RR 1.16; 95% CI 1.08–1.24), patient transporters (RR 1.15; 95% CI 1.09–1.22) and nurses (RR 1.13; 95% CI 1.10–1.15) had a higher risk of infection after adjusting for age and gender. Crude differences in mortality rates were observed according to job category, which could be explained by differences in age, sex, and comorbidity distribution. Diabetes, obesity, hypertension, hemolytic anemia, and HIV were associated with increased fatality rates.

**Conclusions:**

COVID team workers had higher infection rates compared to the total population of active workers and HPW. Doctors had lower risk of infection than respiratory therapists, nurses, and patient transporters, among whom interventions should be reconsidered to reduce risks. The presence of comorbidities, such as diabetes, obesity, arterial hypertension, hemolytic anemia, and HIV, increased the likelihood of complications caused by COVID-19, culminating in a poor prognosis.

On 25 February 2020, the World Health Organization (WHO) (1) declared an international public health emergency due to coronavirus disease 2019 (COVID-19). Given that healthcare workers are the fundamental element responsible for the care of affected patients and control of the pandemic, they may experience increased risk of infection due to contact with infected communities, relatives, friends, patients, or colleagues (2). Therefore, their protection is important for their personal safety, continuation of patient care amidst the health crisis (3), and, most importantly, ensuring that they do not become a source of infection (4–6).

Although healthcare workers account for <3% of the population in the vast majority of countries and <2% in low- and middle-income countries, approximately 14–35% of COVID-19 cases involve healthcare workers (7). This suggests a higher risk of SARS-CoV-2 infection among healthcare personnel given the lack of necessary precautions based on the disease’s transmission mechanism at the beginning of the pandemic (5). Some researchers have reported that there is insufficient evidence indicating that nosocomial COVID-19 infections are the main source of infection among healthcare workers, suggesting the possibility of home environment infections (8).

Therefore, determining whether healthcare workers, especially those in the front line, are at excess risk of infection and death due to COVID-19 is of importance, particularly in developing countries given studies reporting ethnic differences in mortality due to COVID-19 (9, 10). In Mexico, the high prevalence of obesity and diabetes may increase the COVID-19 mortality, especially considering the synergy between the presence of comorbidities, age, and increased exposure.

The Mexican Social Security Institute (IMSS in Spanish) is the largest social security institution in Latin America, covering approximately half of the Mexican population. It provides social, economic, and healthcare services to workers and their families in the private sector of the economy; delivers preventive, curative, and rehabilitation services in 1521 primary-care clinics, 251 second-level hospitals, and 25 third-level hospitals; and employs almost half a million workers, a majority of whom are in healthcare, including groups of doctors and nurses organized into teams for the exclusive care of patients with COVID-19 (COVID teams). During the pandemic, the IMSS provided personal protective equipment to its workers according to the recommendations of the Pan American Health Organization while also maintaining an epidemiological surveillance system of its workers to identify those with COVID-19 symptoms and persons with whom they had come in contact. Thus, this group of workers provides us with a great opportunity to (1) estimate the risks of infection and death by COVID-19 among healthcare workers (2); compare the risks between and within job categories (3); compare healthcare workers, workers in COVID teams, and those under home protection (HPW); and (4) identify risk factors for death among infected workers.

## Methods

A cohort of permanent and temporary workers hired to address the pandemic was followed from March to December 2020. The main outcome studied was RT-PCR-confirmed SARS-CoV2 infection, while the secondary outcome was severe disease including hospitalization and death among these confirmed cases. Cumulative incidence rates were estimated according to job category and activity during the pandemic. The strength of selected risk factors for death was assessed in a group of cases (deaths) and controls (survivors) within this cohort.

### Establishing the cohort of workers

The list of workers was obtained from the IMSS payroll. Job titles were then identified and categorized into three groups: patient care (health workers), administrative workers, and other categories. The job titles considered health workers were doctors, nurses, medical assistants, social workers, chemists/laboratory technicians, histo/cytotechnologists, food handlers, hygiene/cleaning personnel, ambulance personnel, pharmacy staff, radiologists, stomatologists, respiratory therapists, and patient transporters.

### Identifying confirmed cases

Symptomatic cases within all medical units of the IMSS and preventive services for its own workers were identified. Suspected cases were registered in the Online Epidemiological Surveillance Notification System (SINOLAVE in Spanish) and identified by a unique identification number, called CURP in Mexico. In addition to the cases reported in this system, we actively looked for any additional cases reported to the Union and other absenteeism reports to the personnel administrative unit. IMSS workers have economic incentives to avoid absenteeism not related to health conditions; therefore, there is an incentive to report any medical condition that prevents them from going to work.

Workers who satisfied the operational definition of a suspected case of COVID-19 (11) underwent a nasopharyngeal swap to obtain samples for RT-PCR diagnosis and completed a questionnaire for epidemiologic surveillance. A suspected case of SARS-CoV-2 infection was defined as person who during the last seven days presented acute onset of any of two or more of the following signs or symptoms: fever, cough or headache; and one or more of the of the following signs or symptoms: dyspnea, myalgia, sore throat, coryza, diarrhea or conjunctivitis.

Similarly, samples from persons who came in contact with symptomatic workers within their work environment were obtained after four and five days despite having no symptoms.The SINOLAVE registry includes the following variables: age, sex, history of comorbidities, and RT-PCR results. This report included all confirmed cases (symptomatic and asymptomatic) from 1 March to 10 December 2020, as well as all hospitalizations and deaths identified among these cases. We searched four different data sets to look for hospitalizations and deaths among confirmed cases: intensive care unit, emergency room, hospital discharges and SINOLAVE. Emergency room visits were included as hospitalizations if the stay in this area lasted >24 hours during the pandemic.

The samples were processed in the following four reference laboratories: the Biomedical Research Center of the Northeast in Nuevo León, the Biomedical Research Center of the West in Jalisco, the Yucatan Medical Research Unit (UIMY), and the Central Laboratory of Epidemiology in Mexico City. All four laboratories have ISO 9001 certification granted by the Mexican Institute of Normalization and Certification, which is endorsed by the Mexican Accreditation Entity, with a technical capacity endorsement by the National Institute of Epidemiological Diagnosis and Reference.

### Workers under home protection

During the pandemic, the Mexican Ministry of Health authorized workers to abstain from working and granted them stay-at-home protection from 24 March 2020 in the form of a paid leave of absence with benefits for those at higher risk of complications or death by COVID-19 who showed any of the following characteristics: age >65 years, pregnant or nursing women, chronic noncommunicable diseases (arterial hypertension, chronic obstructive pulmonary disease, kidney failure, lupus, cancer, diabetes, obesity, liver failure, and heart disease), or any immunosuppressive disease or treatment. This group of workers, who did not provide healthcare during the leave, were followed up using the same epidemiological surveillance criteria as active workers, representing those with non-occupational risk of infection outside medical care units. This group was used as a control when comparing the risks of infection associated with occupational exposure during medical care.

### COVID team workers

To satisfy the high demand for hospitalized patient care, the IMSS organized health personnel into care teams (called COVID teams) in charge of a maximum of 24 patients. These teams consisted of medical and nursing personnel trained for the direct care of patients with COVID-19 in second- and third-level hospital units (frontline health personnel). A list of workers on these teams available in the SINOLAVE database allowed us to identify infected workers or those who died due to COVID-19.

### Statistical analysis

Illness and mortality risks were estimated as the cumulative incidence rates of infection, mortality, and case-fatality according to job category. Rates were estimated by grouping the workers into the following categories (i): COVID team members (ii), other active healthcare workers (OAHCW), and (iii) workers under home protection (HPW).

Excess risk according to job category was assessed using Poisson regression models with crude and adjusted rate ratios for age and sex. Rate ratios of infection and death due to COVID-19 were estimated according to job category, with administrative workers as the reference category. This evaluation excluded HPW.

The associations between age, sex, and certain comorbidities and the risk of death due to COVID-19 was assessed by comparing survivors and deceased workers using unconditional logistic regression models that compared workers with and without comorbidities with confirmatory tests. HPW were compared to OAHCW, which was the reference category. All analyses were conducted using statistical package Stata version 14.2 (IBM Corp, Armonk, NY, USA).

## Results

### Incidence of infection and mortality due to COVID-19

The IMSS comprised 542 381 workers at the beginning of the pandemic, of whom 41 461 were HPW. Among the remaining 500 920 active workers, 85 477 were doctors and nurses who were members of COVID teams, while 283 884 were OAHCW. The IMSS workers (56.7% female) had a mean age of 37.07 years.

The first imported case of COVID-19 in Mexico was reported on 28 February (12), while the first cases of COVID-19 among IMSS workers were reported in March 2020, with the first death recorded on 29 March.

Among the 500 920 total active workers 30.0% (149 955) reported symptoms consistent with the operational definition of suspected COVID-19, whereas 25.1% (10 414) of HPW sought medical care for similar symptoms. Among total active symptomatic workers, 69 342 were confirmed as COVID-19 cases. Additionally, 1189 asymptomatic workers tested positive for SARS-CoV-2.

Table 1 details the infection, hospitalization, mortality, and case-fatality rates among all IMSS workers, COVID teams, and OAHCW. In general, women had higher infection rates, whereas men had higher mortality rates. The risk of being admitted to a hospital was similar between men and women, increased with age and was higher for HPW. As expected, mortality rates increased with age. Although HPW had the lowest infection rates, they had the highest mortality and case-fatality rates. COVID team members had higher infection rates but the lowest mortality and case-fatality rates.

**Table 1:** Infection, hospitalization, mortality and case-fatality rates in total workers, home protection workers (HPW), active workers and COVID Team workers, IMSS, Mexico, March to December 2020. Sources: Online Epidemiological Surveillance Notification System (SINOLAVE), IMSS; Comprehensive Personnel Management System (SIAP), IMSS. [CI=confidence interval; OAHCW=other active health care workers]

	Workers	COVID-19 PCR-RT test confirmed	Confirmed hospitalized cases	Deaths	Infection per 100 workers	Hospitalization per 100 cases	Mortality per 10 000 workers	Case-fatality per 100 infected workers
							
	N	N	N	N	% (95% CI)	% (95% CI)	Rate (95% CI)	% (95% CI)
Total workers	542 381	75 595	7192	581	13.9 (13.8–14.0)	9.5 (9.3–9.7)	11.0 (10.0–12.0)	0.8 (0.7–0.9)
Men	234 850	32 053	2977	425	13.6 (13.5–13.8)	9.3 (8.9–9.6)	18.1 (16.0–20.0)	1.3 (1.2–1.4)
Women	307 530	43 542	4215	156	14.2 (14.0–14.3)	9.7 (9.6–9.9)	5.1 (4.0–6.0)	0.4 (0.3–0.5)
Age (years)								
≤30	128 947	19 046	1283	14	14.8 (14.5–14.9)	6.7 (6.3–7.1)	1.1 (1.0–2.0)	0.1 (0.04–0.12)
31–45	290 436	41 079	3947	172	14.1 (14.0–14.2)	9.6 (9.3–9.9)	5.9 (5.0–7.0)	0.4 (0.3–0.5)
≥46	122 998	15 470	1962	395	12.6 (12.3–12.7)	12.7 (12.1–13.2)	32.1 (29.0–35.0)	2.6 (2.3–2.8)
HPW	41 461	5 064	658	75	12.2 (11.8–12.5)	13.0 (12.0–14.0)	18.1 (14.0–23.0)	1.5 (1.1–1.8)
Total active workers	500 920	70 531	6534	506	14.0 (13.9–14.1)	9.3 (9.0–9.4)	10.1 (9.0–11.0)	0.7 (0.6–0.8)
COVID team workers	85 477	17 186	1610	42	20.1 (19.8–20.4)	9.4 (8.9–9.8)	5.0 (4.0–7.0)	0.2 (0.1–0.3)
OAHCW	283 884	38 915	3826	333	13.7 (13.5–13.8)	9.8 (9.5–10.1)	12.0 (11.0–13.0)	0.8 (0.7–0.9)

To evaluate the influence of age, sex, and comorbidities on the risk of death among HPW, crude and adjusted (according to comorbidities, age, and sex) odds ratios (OR) were estimated. The crude OR associated with death due to COVID-19 and having remained under home protection was 2.06 [95% confidence interval (CI) 1.62–2.63] when compared to OAHCW. After adjusting for the presence of comorbidities, the OR was significantly reduced (OR 1.63, 95% CI 1.23–2.17), suggesting that this excess risk was partly due to the associated comorbidities and age that justified home protection.

Table 2 summarizes the incidence rates in the working population according to job category, age- and sex-adjusted relative risks for infection, hospitalization and death, with administrative workers as the reference group. Accordingly, respiratory therapists had the highest infection rates (19%), followed by patient transporters (17.5%) and nurses (17.1%). The relative risk for ambulance personnel, social workers, patient transporters, nurses, and hygiene and cleaning personnel remained significantly increased after adjusting for age and sex. Respiratory therapist and patient transporter had increased risk of being hospitalized. Increased mortality and case-fatality rate ratios were observed in ambulance personnel, respiratory therapist, and pharmacy staff, although these relative risks lost statistical significance after adjusting for age and sex.

**Table 2:** Rates and relative risks of infection, hospitalization and death by COVID-19 in active workers by job category. Sources: Online Epidemiological Surveillance Notification System (SINOLAVE), IMSS; Comprehensive Personnel Management System (SIAP), IMSS. [CI=confidence interval; CC=confirmed cases; CHC=confirmed hospitalized cases]

Job category	Workers	CC	CHC	Deaths	Infection per 100 workers	Hospitalization per 100 cases	Mortality per 10 000 workers	Case-fatality per 100 cases	SARS-CoV-2 infection	Hospitalization from COVID-19	Death from COVID-19
										
	N	N	N	N	% (95% CI)	% (95% CI)	Rate (95% CI)	% (95% CI)	RR (95% IC) ^a^	RR (95% IC) ^a^	RR (95% IC) ^a^
Nurse	149 040	25 469	2600	132	17.1 (16.8–17.3)	10.2 (9.8–10.6)	8.8 (7.4–10.5)	0.5 (0.4–0.6)	1.13 (1.10–1.15)	1.52 (1.41–1.64)	1.29 (0.93–1.78)
Doctor	104 306	13 968	1190	139	13.4 (13.1–13.6)	8.5 (8.0–9.0)	13.3 (11.2–15.7)	1.0 (0.8–1.1)	0.91 (0.89–0.94)	1.09 (1.00–1.18)	1.02 (0.74–1.40)
Hygiene & cleaning	30 568	4 501	412	17	14.7 (14.3–15.1)	9.2 (8.2–10.0)	5.5 (3.2–8.9)	0.3 (0.2–0.6)	1.13 (1.09–1.17)	1.29 (1.16–1.44)	0.86 (0.46–1.39)
Medical assistant	28 220	3 741	355	12	13.3 (12.8–13.6)	9.5 (8.5–10.5)	4.2 (2.1–7.4)	0.3 (0.1–0.5)	1.08 (1.04–1.12)	1.29 (1.15–1.46)	0.93 (0.49–1.78)
Chemist/Laboratory technician	12 626	1 849	183	9	14.6 (13.9–15.3)	9.9 (8.5–11.4)	7.1 (3.2–13.5)	0.5 (0.2–0.9)	1.02 (0.97–1.07)	1.24 (1.07–1.44)	0.67 (0.33–1.36)
Food handler	9 297	1 290	116	12	13.9 (13.1–14.6)	8.9 (7.4–10.7)	12.9 (6.6–22.5)	0.9 (0.4–1.6)	1.11 (1.05–1.18)	1.29 (1.16–1.44	1.64 (0.88–3.07)
Patient transporter	8 919	1 561	155	11	17.5 (16.6–18.3)	9.9 (8.4–11.6)	12.3 (6.0–22.0)	0.7 (0.3–1.2)	1.15 (1.09–1.22)	1.54 (1.31–1.80)	1.18 (0.61–2.27)
Pharmacy staff	6 740	961	90	14	14.3 (13.3–15.1)	9.4 (7.5–11.5)	20.7 (11.3–34.8)	1.4 (0.1–2.4)	1.07 (1.00–1.15)	1.16 (0.95–1.42)	1.45 (0.81–2.62)
Social worker	6 094	971	92	3	15.9 (14.9–16.9)	9.5 (7.6–11.6)	4.9 (1.0–14.3)	0.3 (0.1–0.9)	1.16 (1.08–1.24)	1.24 (1.01–1.51)	0.63 (0.19–2.04)
Radiologist	5 106	746	70	8	14.6 (13.5–15.7)	9.4 (7.3–11.8)	15.6 (6.7–30.8)	1.1 (0.4–2.1)	1.08 (1.00–1.17)	1.35 (1.10–1.67)	0.91 (0.43–1.92)
Stoma-tologist	3 641	287	20	2	7.9 (7.0–8.8)	7.0 (4.2–10.7)	5.4 (0.6–19.8)	0.7 (0.1–2.5)	0.91 (0.81–1.02)	0.66 (0.43–1.0)	0.62 (0.15–2.55)
Ambulance personnel	2 677	425	52	12	15.9 (14.4–17.4)	12.2 (9.1–16.0)	44.8 (23.1–78.3)	2.8 (1.4–4.9)	1.20 (1.09–1.32)	1.28 (0.98–1.67)	1.31 (0.70–2.45)
Respiratory therapist	1 336	254	24	3	19.0 (16.7–21.5)	9.4 (6.0–14.0)	22.4 (4.6–65.6)	1.1 (0.2–3.5)	1.10 (0.97–1.25)	1.62 (1.17–2.23)	1.55 (0.48–4.98)
Histo/Cyto-techno-logist	791	78	8	1	9.9 (7.7–12.3)	10.3 (4.4–20.2)	12.6 (0.3–70.4)	1.3 (0.1–7.1)	1.03 (0.82–1.29)	1.55 (0.90–2.69)	0.92 (0.12–6.70)
Other	57 034	5 741	527	76	10.1 (10.0–10.3)	9.2 (8.4–10.0)	13.3 (10.4–16.6)	1.3 (1.0–1.6)	1.06 (1.03–1.10)	1.11 (1.01–1.23)	1.25 (0.88–1.78)
Admin-istrative	74 525	8 689	640	55	11.7 (11.4–11.9)	7.4 (6.8–7.9)	7.38 (5.5–9.6)	0.6 (0.4–0.8)	1	1	1
Total	500 920	70 531	6534	506	14.1 (13.9–14.1)	9.3 (9.0–9.4)	10.1 (9.0–11.0)	0.7 (0.6–0.8)			

a Adjusted for age and sex.

### Risk factors for death

Table 3 presents the risk factors for the development of severe disease that caused death among active IMSS workers who became infected. The presence of certain comorbidities, such as diabetes (OR 2.52, 95% CI 1.94–3.29), obesity (OR 2.05, 95% CI 1.67–2.60), and arterial hypertension (OR 1.30, 95% CI 1.01–1.68, increased the risk of death, while less frequent but very high risk comorbidities included a history of hemolytic anemia (OR10.0, 95% CI 1.20–82.75) and HIV (OR 6.97, 95% CI 1.92–25.28).

**Table 3:** Associations between death by COVID-19 and age, sex, andpresence of comorbidities. [OR=odds ratio; CI=confidence interval;COPD=chronic obstructive pulmonary disease]. Sources: Online Notification System for Epidemiological Surveillance (SINOLAVE), IMSS;Comprehensive Personnel Management System (SIAP), IMSS.

	Surviving	Deceased	Crude OR (95% CI)	Adjusted a OR (95% CI)
Active workers	70 025	506		
Age (per one year)			1.13 (1.12–1.15)	1.12 (1.10–1.13)
Sex (male)	30 112	374	3.82 (3.03–4.80)	2.94 (2.33–3.71)
Presence of comorbidities				
Hemolytic anemia	37	1	6.22 (0.77–50.18)	10.0 (1.20–82.75)
Immuno-suppression	264	14	1.63 (0.59–4.49)	1.62 (0.60–4.33)
Chronic liver disease	56	1	1.86 (0.20–16.57)	0.92 (0.10–7.66)
Obesity	9740	166	1.87 (1.47–2.37)	2.05 (1.67–2.60)
Kidney disease	147	8	1.44 (0.43–4.81)	1.52 (0.44–5.24)
Diabetes	3170	130	4.50(3.45–5.88)	2.52 (1.94–3.29)
COPD	174	8	3.48 (1.53–7.92)	1.48 (0.64–3.44)
HIV carrier	65	5	7.91 (2.14–29.24)	6.97 (1.92–25.28)
Arterial hypertension	6167	161	2.53 (1.96–3.28)	1.30 (1.01–1.68)
Bronchial asthma	2384	19	0.70(0.38–1.31)	0.96 (0.51–1.79)
Cancer	60	1	2.03 (0.28–14.72)	0.26 (0.02–3.37)
Cardiovascular disease	472	10	1.26 (0.56–2.89)	0.66 (0.28–1.53)

a Adjusted for age, sex, and presence of comorbidities.

## Discussion

The current study confirmed that frontline COVID-19 team workers were at excess risk of SARS-CoV-2 infection given their higher risk of exposure to increased viral loads as reported previously (13, 14). However, despite having higher rates of infection compared to other active health workers, they showed no excess risk for hospitalization or death. On the other hand, while HPW were less exposed to infection, they had a higher risk of death once infected. Considering that frontline health workers have a significantly higher risk of COVID-19 infection (14), the ability of WHO-recommended personal protective equipment in mitigating occupational transmission needs more research.

The higher mortality rates among HPW than among other active health workers and COVID teams is expected given that the former had more comorbidities, whereas the latter were healthier and younger. The case-fatality rates found in the present study (0.7% and 0.2% for active health workers and COVID teams, respectively) were similar to those reported in other countries, such as the United States (0.4%) (15), China (0.3%–0.7%) (16, 17), Germany (0.2%–0.5%) (18) and Italy (1.2%) (19).

China reported that women accounted for 79% of infected workers, with the same figure being 57% in Mexico (20). Meanwhile, the current study found that older men had a higher risk of death similar to that reported in China (20, 21).

Several studies agree that health workers directly caring for patients with COVID-19, such as nursing and medical personnel, are at higher risk of infection (3, 19, 21–26). After determining the rates and risks of infection according to job category, the present study found that ambulance personnel, social workers, patient transporters, and nurses were at a high risk of infection, whereas doctors had lower infection rates. Laursen et al (27) reported that ambulance personnel were at higher risk of infection given their increased interaction with patients.

Such differences in the risk of infection according to job categories could be related to not only varying levels of exposures but also heterogeneous precautions taken during their occupational activities, with doctors being more aware of infection risks. Moreover, our results showed that ambulance personnel, respiratory therapist, and pharmacy staff had the highest mortality rates. These findings are in agreement with those presented in other studies, which suggested that while nurses had higher risks of infection, attention should be provided to workers not providing direct medical care to patients with COVID-19 given high risk of infection and death among such workers.

The current study found that comorbidities were prevalent among HPW and were responsible of their greater risk of mortality from COVID-19 compared to active health workers. However, after adjusting for comorbidities, this association was considerably reduced, confirming that isolating these workers in their homes was an adequate strategy for reducing the number of deaths among susceptible workers.

Risk factors with the strongest association included history of hemolytic anemia, obesity, diabetes, HIV, and hypertension, which is in agreement with the findings published in the international literature (17, 21, 28, 29)

This observational study has several strengths and limitations that should be considered when interpreting the results. One of the advantages of the current study is the large population analyzed and the diversity of job categories included. Although an effort was made to include all clinical infections, subclinical infections were not precisely represented. However, bias related to underreporting could be similar across job categories. To assess possible bias introduced by differential testing between occupational categories, we estimated infection and mortality only for symptomatic cases. Rates did not change significantly (data not shown) due to the reduced number of asymptomatic cases; therefore, all results are presented for all confirmed cases independently of symptoms at the time of testing (30–32). An additional limitation could be related to the identification of comorbidities given that these were reported by the worker upon COVID diagnosis.

In conclusion, COVID team workers had higher infection rates compared to the total population of active workers and those under home protection. Doctors had lower risk of infection compared to respiratory therapists, nurses, and patient transporters. As such, interventions aimed at reducing risks among these occupations should be reconsidered. The presence of comorbidities, such as diabetes, obesity, arterial hypertension, hemolytic anemia, and HIV, had been found to increase the likelihood of complications caused by COVID-19, subsequently causing a poor prognosis.
